# Editorial: Stimuli-responsive lipid-bioactive conjugate-based nanocarriers: a smart approach in biomaterial applications

**DOI:** 10.3389/fbioe.2024.1431458

**Published:** 2024-05-30

**Authors:** Ankit Jain, Nishi Mody, Sanjay K. Jain, Umesh Kumar Patil, Abhay Singh Chauhan

**Affiliations:** ^1^ Department of Pharmacy, Birla Institute of Technology and Science-Pilani, Pilani, Rajasthan, India; ^2^ Department of Pharmaceutical Science, Dr. Hari Singh Gour University, Sagar, Madhya Pradesh, India; ^3^ Department of Biopharmaceutical Sciences, Medical College of Wisconsin, Milwaukee, WI, United States

**Keywords:** tumor targeting, nanocarriers, immunotherapeutics, theragnosis, stimuli-responsive, site-directed diagnosis or imaging

Stimuli-responsive lipid-bioactive conjugate-based nanocarriers are a revolutionary approach in biomaterial applications. These advanced nanosystems can remarkably adapt to various stimuli within the body, including pH, temperature, enzymes, and light. Their adaptability empowers precise control over drug release kinetics, minimizing unwanted effects while maximizing therapeutic efficacy. These smart nanocarriers have unparalleled efficiency in traversing physiological barriers and targeting specific cells or tissues, rendering them invaluable assets in drug delivery, imaging, and theranostics. They offer a sophisticated solution for personalized medicine and targeted therapy strategies, which hold great promise in treating various diseases.

Frontiers initiated a Research Topic titled “Stimuli-responsive lipid-bioactive conjugate-based nanocarriers: a smart approach in biomaterial applications” to present a compilation of this Research Topic. This Research Topic is coedited by Dr. Ankit Jain, Prof. Sanjay K. Jain, Prof. Umesh Kumar Patil, and Prof. Abhay Singh Chauhan. Dr. Nishi Mody contributed as topic coordinator.

The special edition of the Research Topic is a sincere and emotional tribute to the impressive and distinguished career of Prof. Sanjay K. Jain, a highly esteemed mentor who has been much more than just a mentor to us. Prof. Jain has profoundly impacted the lives and works of numerous researchers. We will always be grateful for his guidance, which continues to motivate and inspire us in the work we produce and the lives we lead.

The Research Topic received significant interest, resulting in six submissions, of which five were accepted and published, comprising four reviews and one original research article.

The future of treating triple-negative cancer (TNBC) seems promising with advanced research in nano-drug delivery systems. These systems can deliver drugs and immune-enhancing factors to eradicate tumor cells, and their progress looks very hopeful. Doxorubicin, a chemotherapy drug, has shown better efficacy in treating metastatic TNBC. In the coming years, we expect significant improvement in clinical trials combining Doxorubicin with immune checkpoint inhibitors. Zeng et al. have reviewed the progress of nano-drug delivery systems applied to Doxorubicin to treat TNBC and envision developing nanomedicine delivery systems as a new class of safe and effective treatments for TNBC patients. With the continued progress in this field, we can expect more personalized and effective therapies for TNBC patients.

The research conducted by Wu et al. is a notable accomplishment in the field of stimuli-responsive nanomaterials. The comprehensive review not only elucidates the fundamental principles of these materials but also explores their antibacterial capabilities. This study provides valuable knowledge about the potential obstacles to utilizing these materials and possible solutions. The presented information is informative and useful for future research endeavors.

Recently, there has been a new approach to improve the efficacy of anticancer treatments. This approach, called carrier-free chemo-photothermal therapy, has shown much promise. A group of researchers, Kuang et al., have taken this approach to the next level by developing specialized nanoparticles for chemo-photothermal therapy. The study showcases the potential of multifunctional nanosystems in targeted drug delivery and theranostics, a cutting-edge and exciting field of research. This breakthrough holds excellent potential for the future of anticancer treatments.

Nanoagents designed for targeting diagnosis and treatment show promising potential in combating atherosclerosis. In a recent study, Luo et al.emphasized the substantial benefits of using nanoagents, including their ability to selectively deliver drugs to specific sites in the body, reduce drug toxicity, and enhance drug efficacy. The study provides valuable insights into the potential of nanoagents for diagnosing and treating atherosclerosis and highlights their potential in managing various diseases, including atherosclerosis and ovarian cancer.

Zhang et al. conducted a study to develop biosensors that can detect ovarian cancer at an early stage. They used nanomaterials to create these biosensors. The team highlighted the importance of using nanocarriers that respond to stimuli in cancer treatment. This approach can potentially revolutionize current treatment methods and offer more accurate and effective therapies for patients with ovarian cancer. Advancements in diagnosing and treating ovarian cancer can lead to better patient outcomes and improve their quality of life.

In conclusion, all the authors who contributed to this Research Topic have enriched our understanding of stimuli-responsive lipid-bioactive conjugate-based nanocarriers and their transformative impact on biomaterial applications. Their collective efforts have provided valuable insights into the potential of these intelligent nanocarriers in personalized medicine, targeted therapy strategies, and disease management.

